# National shipbuilding strategies in Australia, Britain, and Canada

**DOI:** 10.1177/00207020251320417

**Published:** 2025-02-23

**Authors:** Elinor C. Sloan

**Affiliations:** Department of Political Science, Carleton University, Ottawa, Ontario, Canada

**Keywords:** Shipbuilding, Type 26, warship, naval strategy, Royal Canadian Navy, Royal Australian Navy, Royal Navy, defence procurement, military procurement, naval shipbuilding

## Abstract

This article conducts a comparative analysis of naval shipbuilding approaches by Australia, Britain, and Canada with a view to promoting wider understanding of naval shipbuilding in Canada. Through various strategic and tactical factors, it illuminates how Canadians got to where they are in naval shipbuilding, which processes should be kept, and where there are shortfalls. With a focus on large naval ships, defined as 1000 tons or heavier, the article finds that much of Canada's naval shipbuilding strategy makes sense and should be continued. Yet there are critical problem areas that, if not addressed, threaten to leave the Royal Canadian Navy without the warships it needs.

Much ink has been spilt on Canada's National Shipbuilding Strategy. First announced in 2010, the original strategy identified two Canadian shipyards with which the government would partner for building combat and non-combat ships. In 2011 it announced the successful bids—Seaspan Shipyards for the latter, and Irving Shipbuilding for the former. In 2012 Ottawa signed umbrella agreements with the shipyards.

Progress has been slow. At the Irving yard in Halifax, Arctic Offshore Patrol Vessels (AOPVs) have been built for the navy and two are underway for the Coast Guard, but the River class destroyer (formerly the Canadian Surface Combatant), once promised for the 2020s, will not sail before the 2030s. At the Seaspan yard in Vancouver, fisheries vessels have been delivered to the Coast Guard, but two navy supply ships, originally planned for the early 2020s, are now expected to be delivered in 2025 and 2027 at the earliest, and work will not begin on a polar-class ice breaker before the end of the decade.

Critics have decried the delays and the attendant cost increases, regularly reported on by the Parliamentary Budget Office and others. Yet this information does not promote wider understanding of naval shipbuilding in Canada, nor illuminate how and why we got to where we are, which processes should be kept, and where there are shortfalls. This article fills these gaps. It provides a comparative analysis of strategic and tactical shipbuilding approaches used by Australia, Britain, and Canada. With a focus on large naval ships, defined as 1000 tons or heavier, the article argues that much of Canada's naval shipbuilding strategy makes sense and should be continued. Yet there are critical problem areas that, if not addressed, threaten to leave the Royal Canadian Navy without the warships it needs.

## National shipbuilding strategies in Canada, Australia, and Britain

Between 2010 and 2017 Australia, Britain, and Canada each announced a naval shipbuilding strategy. Canada was first out of the gate with its 2010 National Shipbuilding Procurement Strategy, renamed the National Shipbuilding Strategy (NSS) when the Justin Trudeau government came into office in 2015. Australia launched a Naval Shipbuilding Plan in May 2017, and Britain followed in September 2017 with its National Shipbuilding Strategy.

All three strategies were driven by escalating costs and delays in producing large naval ships. For Canada, the spark was the government's 2008 decision to cancel projects to replace Navy supply ships and Coast Guard mid-shore patrol boats because the bids were considered too high. Plans for the Single Class Surface Combatant were also shelved in 2008, only five years after the cancellation of an earlier destroyer program, the Command/Control and Air Defence Replacement, which had been in the works since the mid-1990s. For Britain, the instigating factor was that in the early 2010s it was caught in a downward spiral where fewer ships were being built at ever greater cost. And Australia's concern was its Air Warfare Destroyer program was experiencing serious cost overruns, delays, and productivity problems. Moreover, all three countries knew that addressing the current ship at hand would not resolve the naval shipbuilding problem because each had several fleets of ships that needed to be built over the subsequent decades. What was to be done?

## Strategic considerations in naval shipbuilding

### Warship building and sovereignty/self-sufficiency

The starting point in understanding the wider picture of naval shipbuilding in these countries is the notion of sovereignty or, at a minimum, self-sufficiency. Both perspectives, closely related, lead to the perceived necessity of building warships at home. In Canada the sentiment dates to the First World War, when it became apparent how dependent it was on Britain for acquiring naval vessels. After the war, government officials argued that naval shipbuilding had to be a national undertaking for Canada, given its extensive coastline and dependence on overseas trade.^1^ From the Second World War onward it was Canada's policy to build warships at home.^2^ This was codified in a 2001 build-in-Canada policy decision,^3^ later reaffirmed in 2007 under the Stephen Harper government.^4^ Since 2010, through the NSS, the Government of Canada has committed to building all naval vessels in Canada.

Sovereignty or self-sufficiency is also part of Australia's dialogue. Its 1976 Defence White Paper spoke of the need for self-reliance. Although this was not tied explicitly to shipbuilding, in subsequent decades most Royal Australian Navy ships were built in-country. Australia's 2017 naval shipbuilding program was explicitly presented as a means of generating a sovereign shipbuilding capability in Australia.^5^ That said, the country's defence department has questioned the wisdom of building naval vessels in Australia, arguing that there is no strategic reason to build warships in Australia.^6^ The perspective reflects the fact that historically, Australians have not had concerns about procuring non-Australian designs, even as their defence strategies have stressed the need for self-sufficiency in fleet production and maintenance.^7^

Finally, Britain is also compelled in its shipbuilding approach to build warships at home. Historically doing so was a necessity as the preeminent industrial power, although during the Second World War many of its warships were built in Canada. During the Cold War, world-class shipbuilding capabilities emerged in many places, notably the Far East and especially South Korea. Despite this pull, Britain's 2005 defence industrial strategy called on Britain to retain a sovereign UK warship building industry. An independent shipbuilding report commissioned by the government in 2015 stated that all Royal Navy warships should be built in the UK for reasons of national security.^8^ The position was adopted wholly in the UK's subsequent National Shipbuilding Strategy.

There is a distinction among the three countries, however, on the approach adopted for naval vessels that are not warships. Canada has always built its supply ships at home. Its new Protecteur class Joint Support Ships are being built in Vancouver, and its Auxiliary Oiler Replenishment vessels were built in Saint John, New Brunswick in the early 1970s. Yet Britain and Australia have a different historical practice. UK policy allows for large, non-complex naval vessels, those that are not classified as warships and filled with advanced and sensitive technology, to be procured abroad. Britain's Tide class navy tankers, for example, were built in South Korea. Australia's two amphibious assault ships were built in Spain to the level of the flight deck, and its Auxiliary Oiler Replenishment ships were also built in Spain before being floated to Australia for final fit-out.

One might reasonably ask why self-sufficiency in naval shipbuilding is so important for Canada. Why does it buy fighter jets from the United States and tanks from Germany, but has never looked abroad for its ships? Psychological explanations include an intangible yet important link between naval shipbuilding and Canada's growth as an independent nation, making shipbuilding an expression of political independence and national identity.^9^ After the Second World War Canada also saw warship construction as an opportunity to revitalize Canadian industry.^10^ The St. Laurent class contracts were allocated to ensure shipbuilding capability in key strategic regions of the country, and regional benefits figured in awarding contracts for the Iroquois class and, later, the Halifax class. “After 1977,” writes naval historian Marc Milner, naval fleets were no longer “pitched on their military merits” but rather “on their political and economic value.”^11^

### Sovereignty at stake?

Thus, although sovereignty or at a minimum self-sufficiency is important, the commitment to building naval vessels at home over buying from abroad also relates to job creation and skills development. More than a plan for building new ships, naval shipbuilding in Canada is considered an investment in its industrial and technological capabilities, designed to ensure that the country writ large develops engineering and technical skills. The Conference Board of Canada, for example, projects shipbuilding at the Irving shipyard in Halifax will have added almost $12 billion to Canada's GDP between 2013 and 2025 and sustained an average of more than nine thousand jobs across Canada.^12^

Similarly, a 2015 report on Australia's naval shipbuilding enterprise argued a robust shipbuilding industry would create a skilled-labour pool that could spill over into other venues, adding some two thousand jobs to local economies.^13^ The Shipbuilding Plan itself includes an entire section on building and sustaining a naval shipbuilding workforce. The creation of thousands of jobs in Australian industry continues to be a central selling point for building and upgrading naval capabilities.

Finally, historically, Britain has been concerned with maintaining high-value skills in Britain through warship building, and in recent years has stated that “social value” is an important element of its naval strategy. Its National Shipbuilding Strategy refresh includes a Social Value Model that says the government will consider supply chain competitiveness, skills development, representation of underrepresented groups, and the economic impact on local communities, among other things, when it comes to evaluating bids.

### The build-at-home premium

At the heart of the matter are two questions, both of which elude definitive response. First, accepting that it is more expensive to build ships at home than to buy abroad from an established shipbuilder, how significant is the delta? As far back as the Second World War it was assessed that shipbuilding in Canada came with a 30 percent premium over building in Britain.^14^ More recently, it is thought that Canada's two Joint Support Ships will cost two and a half times that of Britain's four new navy tankers built in South Korea,^15^ and its AOPVs have cost far more than similar vessels in other countries.^16^

But the true delta between build-at-home and buy abroad is imprecise and may be overstated once all factors are considered. A 2008 internal audit by the Department of National Defence found that the build-at-home premium for the Halifax class frigates amounted to only 7 percent, well offset by the many jobs created, while a 2009 Industry Canada report found that, due to required in-service support costs once delivered, foreign builds came with a 25 percent increase.^17^ The long-term savings in building ships abroad are not always clear once intellectual property transfer costs are factored in. There has been no study in Canada designed to establish the build-at-home premium, but in Australia the 2015 report noted above concluded that the domestic production of naval ships carried a price premium of between 30 and 40 percent.

Second, what would be the net economic benefit to building abroad and using the savings to invest in creating other domestic jobs and skills? Savings achieved by building abroad could be outweighed by the loss of national economic benefits, especially when one accounts for the entire lifecycle of a vessel dependent on a supply chain and workforce external to the country. Alternatively, the net economic loss or benefit could be minimal. Some have questioned whether there is a direct relationship between government support of shipbuilders and the actual creation of jobs, since labour is a cost of production, something efficient producers try to minimize in order to maximize profits.^18^ From this perspective, a government could just as well use its resources in other sectors to benefit industry and the economy.

In the end, building most (Australia and Britain) or all (Canada) large naval vessels at home is a strategic choice that has been made by all three of the countries examined here. Assuming build-at-home, how, then, to achieve value for money?

### Continuous build

All three countries examined here have adopted a continuous-build approach as the best means of producing affordable large naval ships in a timely manner. The idea is to avoid a wasteful “boom-and-bust” cycle of shipbuilding in which a shipyard modernizes its facilities and hires and trains a large workforce for a major government contract, but once the contract ends, the yard scales back its workforce and lets its shipbuilding capacity decline to the point that it must be recreated for the next job. A continuous-build approach is thought to be more cost effective in the long run than restarting production at a dormant facility, with all the associated infrastructure and skilled labour costs, every time a new class of ship is to be built.

The key is regular, continuous work. There is a minimum tempo of construction necessary to obtain efficiencies. At a certain point, the cumulative effects of learning among shipyard workers reduce the cost of labour for successive builds of the same ship and even for the set-up costs for building new designs. Australia has identified a “drumbeat rate” of two years for its Hunter class frigate, meaning twenty-four months between the start of construction of each ship.^19^ Britain, too, references continuous build and “drumbeat” in the context of value for money: It is “the rhythm of production of ships, to keep the workforce busy and efficient, like you’d find on a factory production line.”^20^ Experts inside and outside the UK ministry of defence consider a regular drumbeat of design and manufacturing work to be a prerequisite for driving productivity.^21^

### Continuous build with shipyard rationalization

Logically speaking, a continuous-build strategy will require shipyard rationalization or consolidation if there are numerous yards in existence. In the decades after the Second World War, Canada had perhaps half a dozen naval shipyards in operation at any one time. But by the early twenty-first century many of its allies had developed strategic sourcing arrangements with domestic shipyards to build and maintain their military ships. Britain, for example, had such an arrangement in place with a single national shipbuilder. In 2010, with a desire to rationalize the number of yards and eliminate “boom and bust,” and given geographical and regional considerations, Canada decided to establish a strategic sourcing arrangement for large naval vessels with two shipyards, one for combat ships and one for non-combat vessels.

Uniquely amongst its allies, Canada ran a competition to determine the two shipyards, including a request for proposals, bid submission, and evaluation procedure akin to a major military procurement. Although each of the Irving and Seaspan yards won a portion of the competition, a third yard, Chantier Davie, also submitted a bid. Emerging from bankruptcy protection at the time, it was the yard not chosen. In 2019, with a growing backlog of ships, the government launched a competitive process to select a third strategic shipbuilding partner. Four years later Chantier Davie, its finances now in order, was confirmed as the third strategic partner, marking the end of the yard's exclusion from the National Shipbuilding Strategy. Views are mixed on whether continuous build will be sustainable in a three-shipyard scenario, and therefore whether future shipyard rationalization will be necessary in Canada.

Shipyard rationalization also figures in Australia's naval shipbuilding approach. One independent study examined the number of naval shipyards the country could sustain under a continuous build strategy, concluding that “it would be difficult and costly to sustain more than two shipyards.”^22^ Indeed, the country would be challenged to sustain more than one domestic shipbuilder of large warships, but to mitigate risks, it was better to maintain a second yard. Incorporating the report's advice, Australia's shipbuilding plan includes two main yards: Osborne Naval Shipyard in South Australia for major surface combatants and submarines; and Henderson Defence Precinct in Western Australia for minor naval vessels. Taken without great fanfare, the decision required some shipyard rationalization in that prior to the creation of its plan Australia had four major shipyards with a role in naval shipbuilding.

### Continuous build with multiple yards (distributed shipbuilding)

Britain's 2017 National Shipbuilding Strategy adopted a different approach from Canada and Australia. The strategy outlines a plan to *reverse*, at least tentatively, a process of rationalization that it had pursued for many years and that had culminated, in 2009, in a terms of business agreement that legally bound Britain to provide BAE Systems Maritime – Naval Ships with fifteen years of shipbuilding work. Britain had been trying to restrict warship building to just three yards for some decades,^23^ but it was not until the late 1990s that the goal was achieved.^24^ Even then, governments continued to press for further consolidation. After BAE, which has two surface warship yards on the Clyde River in Glasgow, bought out VT Group in Portsmouth in 2009, warship building in Britain lay in the hands of just one company.

It was thought that this new situation of having one surface ship supplier, BAE, and one customer, the Royal Navy, would lead to shipbuilding efficiencies. Yet while consolidation did produce savings for the defence ministry, it may also have stifled innovation, resulting in the downward spiral that sparked the 2017 shipbuilding strategy. A 2016 independent report into Britain's naval shipbuilding recommended breaking up this “closed-loop” system by distributing shipbuilding around Britain.^25^ The Type 26 frigate was already set to be awarded to BAE and its yard on the Clyde, but for the upcoming Type 31 general-purpose frigate, the report argued, the government should adopt a “virtual shipbuilding model” whereby fully outfitted “blocks” or sections would be built at several yards that had demonstrated their cost competitiveness, before being brought together for assembly in a central location.

Britain's Naval Shipbuilding Strategy incorporated the distributed build model. At the same time, its drafters remained cognizant that the model could bring higher integration costs. A report that evaluated the Type 45 destroyer some decades earlier had cautioned a multi-yard approach brought several risks, including potential misalignment of blocks.^26^ This was exactly the issue Australia faced with its Hobart class Air Warfare Destroyers when it used the distributed block approach. Hull blocks for the forward superstructure, remaining superstructure, keel, and sonar were built in Australian yards at Adelaide, Newcastle, Williamstown, and in Britain and Spain, with final integration set for shipbuilder ASC in Adelaide. In 2010, after the block construction began, it was revealed that the central keel block of the first in-class Hobart was distorted and did not fit the other sections of the warship. The situation contributed to an almost three-year delay in delivery of the Hobart class to the Royal Australian Navy.

As for Britain's Type 31, the Ministry of Defence said it would “test the benefits of the virtual shipbuilding model against the single yard build model during the *Type 31e* procurement.”^27^ In 2019 the contract for Type 31 frigates was awarded to Babcock. Construction of blocks is spread between yards across the UK, with final ship assembly at the Babcock yard at Rosyth. There are signs that this will be a successful approach: the keel for the first Type 31 was laid down in 2022 and the ship is expected to be fully in service with the Royal Navy by 2027, followed by the others in the class within a few years.

For all future surface ship procurements Britain plans to consider both distributed builds (across yards) and traditional builds (in one shipyard). Australia, by contrast—after the experience of the Air Warfare Destroyer—has rejected a distributed block-build strategy in favour of vertically integrated construction at a single yard. Canada has never done block builds in separate yards for subsequent integration in a main yard, and has no plans to do so under the current shipbuilding strategy. It has only undertaken distributed construction in the sense that entire ships of one class have been allocated to different shipyards, most notably the Canadian Patrol Frigate. It may be that distributed block build is best suited to geographically small countries like Britain.

Whether building in one yard or across yards, all three countries have adopted block build approaches in their naval shipbuilding. Not long ago, Britain contemplated whether it would be best to employ a whole ship-build method, meaning a traditional keel-up, one ship at a time way, or alternatively a block build approach. Building in blocks allows for the parallel construction of ships, potentially speeding up the process. The method was first introduced in Japan in the 1960s and later perfected in South Korea and in Europe. Australia's Anzac frigates were built in blocks in Australia and New Zealand and integrated in Australia; the UK's Queen Elizabeth class aircraft carriers were built in super blocks across several UK yards for integration at Babcock in Rosyth; Canada's Joint Support Ships are being built in blocks at a single yard, as were the Coast Guard's offshore fisheries vessels; and, blocks for Australia's Hunter class frigate are being built by ASC at the Osborne naval shipyard for integration at that same yard.

### Continuous build—value for money?

Continuous build to avoid boom and bust has been touted as a means of achieving value for money over the long term in naval shipbuilding. Yet there are some important downsides. The promised higher efficiency and productivity of such a program must be balanced against the cost of more frequent vessel replacement and perhaps unnecessary fleet expansion. Two of the countries examined here have had to undertake “gap-filler” builds. When Britain's Type 26 ended up being delayed, to keep the BAE yard in work the government was compelled to order two more offshore patrol vessels, ships “that the Royal Navy neither wants nor knows how it will crew.”^28^ Similarly, delays in the construction of its warship fleet, the River class destroyers, meant that in 2019 Canada had to order two AOPVs for the Canadian Coast Guard to avoid a gap in the continuous build cycle at the Irving yard in Halifax. As a result of a new defence policy that focused on sovereignty in the Arctic, construction of the AOPVs had been prioritized in the build sequence over recapitalizing the navy with a major surface combatant suited to its traditional doctrine of blue water international operations.

But the greatest economic challenge with a continuous-build program is how to ensure efficiency in a monopoly situation. Some have argued that the entry barriers for new firms in so complex an industry as naval shipbuilding is such that competitive tendering is difficult, if not “logically impossible,” while others have disputed the wisdom of continuous, guaranteed shipbuilding work, arguing that the basic economic principles of competition should be applied to the shipbuilding sector.^29^ Shipbuilding as a regional benefits program is an important backstory to all of this and directly impacts how it is carried out. Prior to Canada's 2011 strategic partner decision, Nova Scotia and British Columbia lobbied heavily for the Irving and Seaspan yards, respectively, in a competition that pitted the East Coast, West Coast, and Quebec (home to Davie shipbuilding) against one another. Britain's approach, too, has been to deliberately spread shipbuilding work around the country to small and large yards alike. The regional benefits imperative is not so dominant in Australia but can still be seen in the decision to maintain a major yard at Perth rather than fully consolidating the work at the Osborne Naval Shipyard in South Australia.

Canada has tried to incorporate market-based elements into its shipbuilding enterprise by running a competion for which yards would be strategic partners in providing combat and non-combat naval vessels and establishing thirty-year umbrella agreements with these yards, from 2012, which state that being a strategic partner does not guarantee a contract to build ships. Rather, each contract is negotiated with a prospective yard under parameters set by the umbrella agreements, and each major phase of a shipbuilding project is also subject to a separate contract. Thus, for example, the federal government has negotiated several contracts with Irving to build the River class destroyer, such as a $185 million design contract in 2019, but it has not yet awarded Irving a contract to build the actual ships, though an agreement is expected in 2025. A similar umbrella agreement has now been established with the third shipyard.

Experts also consider financial transparency and open-book accounting throughout the negotiation and building period to be important in regulating a monopoly supplier.^30^ But, in essence, there is no good answer as to how to square the circle between having one builder and achieving value for money. The government has limited recourse should timelines slip and costs increase. The British Ministry of Defence has stated that its continuous shipbuilding pipeline cannot endure if the industry fails to demonstrate efficiency and productivity.^31^ Yet the alternative is to return to the traditional method of the yard and builder competing for each contract, leading to unaffordable bids by virtue of having to price facility upgrade costs into a contract on each occasion.

### Continuous build and infrastructure

An advantage of continuous build is that it can eliminate the need to recreate the necessary infrastructure for large vessel construction every time a new fleet is to be built. Canada launched its national shipbuilding strategy a decade and a half after any naval shipbuilding had taken place in the country, and almost thirty years after it had built any large coast guard ships. The interlude led to the requirement for significant infrastructure investment ($300 million for Irving and $200 million for Seaspan) and a delay of about three years before the yards could start building ships. “Not for the last time in Canadian naval history,” Mark Milner wrote of Prime Minister Wilfrid Laurier when establishing the Canadian navy, “the government realized that, to build a fleet at home, it first had to build a shipyard.”^32^ A century later this still rings true. Capacity remains a major risk at Canadian shipyards and the federal government has allocated millions of dollars for upgrades at Seaspan and Irving. In 2023, for example, Canada invested $463 million in the Irving infrastructure to ensure that the yard can build the future River class destroyer.

Australia by contrast, has incorporated infrastructure upgrades directly into its shipbuilding strategy, stating up front that shipyard infrastructure upgrades would be completed at the Australian government's expense. An entire chapter of the strategy is devoted to government initiatives to upgrade shipbuilding infrastructure at the two designated yards. The math comes down to whether it is the country or the shipyard that must absorb the costs of getting a naval shipyard to what is known in the international shipbuilding industry as “target state.” Although in a competitive environment a case could be made that it is the shipyard's responsibility, at the same time the government may end up with unpalatable bids because the high cost of infrastructure upgrades is included in them. Or, motivated to win a contract, a shipyard might promise more than its infrastructure can deliver, creating downstream risks to project timelines and completion. It is not difficult to see how a strict focus on continuous build and shipyard rationalization can help address these dilemmas.

## Building the ship—tactical considerations in naval shipbuilding

### Modularity

At the more tactical level of naval shipbuilding, experts argue that navies should focus on modularity in future warship design.^33^ Modularity can be defined as “the ability to reconfigure a warship for various missions by loading capability (i.e., modules) in and out of a common frame (i.e., a standardized ship hull).”^34^ Examples of modules for warship missions might include those for anti-submarine warfare, mine counter measures, anti-surface missiles, and autonomous systems, while those for coast guard vessels may be for things like search and rescue and oceanographic survey.

Modularity in warship design is being driven in part by the increasing cost of permanent-fit platforms, which has prompted naval forces to look at other options. Britain's shipbuilding strategy views modularity as central to its vision for future Royal Navy ships since modularity allows for flexibility in the capability choices that are made for major naval platforms.^35^ The mission module approach is also being driven by the need to accommodate the rapid pace of technological advances. Having capabilities within removable modules may enable regular technological upgrading of sensors and weapons systems, and the integration of entirely new capabilities that may not be mature enough to include during the ship's original construction, for example, autonomous systems.

Modularity in naval shipbuilding is starting to become mainstream. Both the Type 26 and Type 31 have been designed to accommodate mission modules that can be loaded into flexible mission bays. Taking the Type 26 as their reference design, both the Hunter class and the River class have also incorporated modularity. Indeed, the modular approach to warship design has been Canada's intent since well before the Type 26 was in consideration.^36^ A least one analyst has questioned the value of modularity, noting that the limited historical experience so far indicates mission modules are rarely, if ever, switched out.^37^ Nonetheless, mission modularity can, in theory at least, offer a means of responding to the rapidly changing technological landscape.

### Design and build

In naval shipbuilding the ship designer must work closely with the ship builder. This lesson was most glaringly on display in Australia, where the ship designer for the Air Warfare Destroyer, Navantia, was not part of the alliance of shipbuilders that built the ship. Different interpretations of the design emerged across the multiple yards and shipbuilders, with the result that when they arrived at the consolidation yard, ASC, some of the blocks did not fit together. Because the contracting model for the air warfare destroyer put the designer at arm's length from the shipyards constructing the ship, the designer was not invested in the outcome of the build.

Careful not to repeat the mistake, Australia set up the build for the Hunter class frigate to ensure the close integration of warship designer and builder. Whereas for earlier frigate and submarine builds the designer was subcontracted to the shipyard, in the Hunter's case the shipbuilder, ASC, has become a temporary subsidiary of the designer, BAE. Canada has not had to resort to so drastic a measure, but it is a significant point that the shipyard that is building the River class destroyer, Irving in Halifax, is working closely with the combat systems integrator, Lockheed Martin Canada, partnered in turn with the warship designer, BAE. Under a design-and-build approach, the shipyard has been able to refine the design before negotiating the build contract.

### Mature reference design

Efficient naval shipbuilding is dependent on the maturity of the design at the start of construction. Part of the problem with Australia's Air Warfare Destroyer was that even as the work was progressing in various yards, the design had not been finalized. Along similar lines, in 2018 Australia and Canada selected BAE's Type 26 Global Combat Ship as the reference design for their Hunter class frigate and River class destroyer, respectively, even though the Type 26 only existed on paper. Unlike designs put forward by Spanish shipbuilder Navantia and Italian shipbuilder Fincantieri, based on ships already in service with their navies, the Type 26 was a new design, with only one ship under construction at the time and no ships in the water.

Both the Canadian and Australian decisions sparked political and media controversy. When it entered office in 2015, the Canadian government specified that the new warship must be based on a proven foreign design. Yet only a year later it prequalified BAE's Type 26 Global Combat Ship, amongst other ship designs. In explaining its about-face, the government argued that since any bid would need substantial Canadian modifications, every bidder could be seen, in essence, as offering a “paper ship.”^38^ But the true reason may have been the lack of shipbuilding capacity and competence within the government, lost in the years since the Canadian Patrol Frigate (CPF) program ended. Although in 2011 Irving won the strategic partner role for combat ships, the question remained: Who in government would lead the competition? As a high-ranking naval officer explains:

A key difference between 2011 and the CPF was the overall capacity within government. All were consumed with getting on with the program. There was no discussion about rebuilding the shipbuilding capacity that Canada had had in the 1980s. Irving started to fill that space. By the time we got to 2015 it was clear the government would need to rely on Irving [a shipyard with a longstanding relationship with BAE].^39^

Australia, too, had committed to a mature design in its shipbuilding plan, yet chose BAE's Type 26 over the ships on offer by Spain and Italy. Here, the decision may be explained by a combination of BAE's longstanding heritage in the country, its expertise in anti-submarine warfare, and Five Eyes intelligence considerations.^40^ Relatively uncontroversial when announced in 2018, the decision sparked media and government debate within a few years. A 2021 report from the Australian defence department's engineering team, leaked in 2022, found serious problems with the “immature” British design.^41^ Although BAE disputed the suggestion of delays in the design, British parliamentary hearings in 2023 revealed that the Ministry of Defence itself felt it had been overly optimistic in its forecast for completing the engineering design for the Type 26.^42^ Other studies have questioned the value of the “modified parent-design” approach altogether, arguing a number of naval ships based on a parent design—mature or not—have experienced unexpected cost and schedule growth.^43^

### Bespoke requirements

It is tempting to blame shipbuilding delays on the lack of a mature and proven reference design. Yet the fact is that while construction of blocks that will be used in the first Hunter class frigate started only in late 2023, and in the River class in mid-2024, steel was cut for the lead Type 26 ship *Glasgow* at the BAE yard on the Clyde back in summer 2017. Clearly something else is amiss. Scholars have convincingly argued that for successful procurement and implementation to occur, there must be long-term historical policy alignment between a country's military force and government.^44^ At the more tactical level, a central issue lies in country-specific design changes.

Up to the flight deck level, Canada and Australia intend to keep the River and Hunter classes essentially unchanged from the Type 26 blueprints. But there are many bespoke topside changes. The biggest areas of adaptation for the River class are those to accommodate Canada's unique command management system, Lockheed Martin's CMS 330; the US Navy's Aegis combat weapons system, which integrates radar information with missiles to intercept threats; and the radar itself, which is to be Lockheed Martin's AN/SPY 7. Australia is also integrating the Aegis system, as well an indigenous phased-array radar from CEA technologies, and a Saab-Australia combat system to interface with Aegis. The list of specific Australian changes that BAE must make to the Type 26 parent design runs to six broad categories.^45^ In Canada it has been reported that of the twenty-six major systems on the River class, nineteen have needed platform changes.^46^

All this takes design time. In 2019 the Government of Canada and Irving Shipbuilding awarded a sub-contract to Lockheed Martin Canada to customize the Type 26 design to accommodate Canada's unique military and industrial requirements. This ongoing phase of “requirements reconciliation” involves adjusting the design of the ship that Britain is sailing to that which Canada will sail, switching out systems or upgrading aspects that do not meet Canadian specifications. A similar process has been underway in Australia, where a 2023 auditor general report found (Australian) design immaturity to be the source of delays in the project.^47^

There are strategic implications to some of these bespoke elements. The Aegis combat weapons system being integrated on the Hunter and River classes also comes in an expanded BMD version. Aegis BMD deployed on US ships is the sea-based leg of America's ballistic missile defence system, designed to strike ballistic missiles in their mid-course phase. Although it has been involved in detecting ballistic missiles since 1960, Canada has historically refused to join the response component of BMD. Any move towards Aegis BMD would therefore be of strategic importance.

Eschewing bespoke requirements is a common aspiration. A guiding principle of Australia's shipbuilding plan was to limit the number of unique Australian design changes.^48^ Similarly, Britain's shipbuilding strategy states that in developing its various classes of ships the Royal Navy will keep bespoke military capabilities to a minimum.^49^ In Canada there is no such promise, primarily because the country's shipbuilding strategy does not exist as an actual document. Early on, however, there was indication that when it came to the River class, government officials were looking for a proven ship design that could be modified for Canadian needs.

Relatedly, a British parliamentary report from 2023 advises against “exquisite procurement,” that is, designing new equipment with requirements that seem to want everything, are unnecessarily complex, and therefore lead to delays, budget pressures, and ultimately fewer platforms.^50^ Both the Hunter and River classes can be seen in this light. Britain's Type 26 is a frigate; it will sail with the Royal Navy's existing and relatively new Type 45 air defence destroyers, as well as the eventual Type 83, an air defence variant of the Type 26. By contrast, both the Hunter and River classes are a combined frigate and destroyer and thus take on significantly more tasks than the reference design. Both combine an area air defence capability with anti-surface and anti-submarine warfare capabilities. Moreover, while the Hunter class will sail with Australia's Hobart class Air Warfare Destroyers, the River class, which has been officially designated a destroyer, will also take on the command and control of other ships.

Undertaking so many roles with one vessel creates design challenges, not least of which is accommodating all the necessary components on the platform. “It's an open question whether the Type 26 hull can support the radar and missiles that a destroyer would require,”^51^ one report observed. Topside changes to the design are adding significant weight to the platform and have necessitated making the hull longer. With an initial planned weight of 8,800 tons, the Hunter class is now on track to exceed 10,000 tons. This is acceptable if it does not impact performance in terms of speed and stealth, but the leaked engineering team report noted above raised some red flags in this area.^52^ In Canada, too, the River class has grown bigger and more complex than originally envisioned; this was the source of the requirement to provide Irving with additional infrastructure funding in 2023. Writing in 1983, Norman Augustine determined that the unit cost of military equipment was increasing at an exponential rate with time, at a factor of four every ten years.^53^ He predicted that as platforms became ever more complex and expensive, militaries would be forced to cut the number they bought.^54^ To date the Canadian government has resisted the Augustine prediction, staying firm at fifteen as the number of destroyers it will buy. Australia, however, has already cut back its Hunter class order from nine to six ships.

## Conclusions

Building large naval vessels, especially a warship, is an exceedingly challenging undertaking. In the second decade of the twenty-first century, Australia, Britain, and Canada each embarked on a path to recapitalize its fleet. Mid-way through the third decade, this remains a story of delays, escalating costs, and fewer ships in the water than had been originally planned for. From the outside, it seems an exasperating and inexplicable situation. Examining the naval shipbuilding endeavour through the lens of various strategic and tactical factors can provide, if not answers, at least a wider perspective on why we are where we are, which processes have made sense and should be kept, and where there must be adjustments.

Naval shipbuilding is much more than a business venture. It is tied closely to the notion of sovereignty or at a minimum self-sufficiency. This can be seen most predominantly in the Canadian case, but also in Britain, which while open to building non-warships abroad builds its warship at home, and in Australia, where after 1976 self-reliance became a main strategic consideration, placing pressure on the government to maintain a long-term shipbuilding capacity.^55^ Naval shipbuilding is also tied to the desire of governments to invest in a highly skilled labour force. The ability of shipbuilding to create thousands of highly skilled jobs, and support domestic industry, is a central explanation for Canada's longstanding naval shipbuilding approach.^56^ A similar theme, to lesser degree, can be seen in Australia and Britain.

At the same time, governments want to produce affordable naval vessels in a timely manner. Strategically, a continuous-build strategy with shipyard rationalization to a minimum of two yards—and investing in shipyard infrastructure—is a logical long-term approach, as is block-build in a vertically integrated yard. Boom and bust in warship building has proven detrimental in the past, and seeking to overcome it is a sound approach. Yet it is difficult to hold a monopoly builder to account. One way of doing so, admittedly unsatisfactory, is for the government customer to mandate and enforce financial transparency and open-book accounting throughout the design-and-build period. Britain has also attempted to add market competition to its process of warship building by asking three UK teams to compete for the Type 31e, resulting in the Babcock win.^57^ Building “non-warships” offshore continues to be an option, although factors like guaranteeing supply chains and maintaining intellectual property make it difficult to ascertain the final savings.

At the more tactical naval shipbuilding level, modularity appears a promising means of responding to the ever-changing and advancing technological landscape. There must also be a close working relationship between the ship designer and the ship builder throughout the build. The critical stumbling block remains what many have known all along: the imperative of choosing a mature, in the water design, and—critically—resisting to every reasonable degree the urge for bespoke requirements. The key is to address an ingrained military culture that lends itself to pursuing excessive complexity in naval shipbuilding. The St. Laurent class ships that Canada built in the 1950s were not just highly capable vessels but “‘Cadillacs’ … the best anti-submarine frigates in the world.”^58^ They cost three times as much as originally projected and were commissioned several years behind schedule. Three quarters of a century later, the lesson is still being learned.

## Notes

1. Alexander Johnston, “The shipbuilding situation and outlook in Canada,” *Canadian Railway and Marine World* (March 1918): 120.

2. Martin Auger, *The National Shipbuilding Procurement Strategy: A Five-Year Assessment* (Ottawa: Library of Parliament, 2015), 1.

3. Tom Ring, *The National Shipbuilding Procurement Strategy: How Did We Get to Where We Are Now?* (Canadian Global Affairs Institute, March 2016), 10.

4. “National shipbuilding procurement strategy,” Office of the Auditor General of Canada, Ottawa, 2013, 5.

5. Rear Admiral Tony Dalton, testimony before the Australian Parliament's Foreign Affairs, Defence and Trade Legislation Committee, 8 November 2018. I am grateful to PhD student Will Richardson for research assistance in identifying this testimony.

6. Gregory Copley, “The future of Australian naval shipbuilding,” *Maritime Studies* (March–April 2006), 1.

7. I am grateful to an anonymous reviewer for this observation.

8. John Parker, “An independent report to inform the UK National Shipbuilding Strategy,” UK Ministry of Defence, London, 29 November 2016, 12.

9. Garth Wilson, *A History of Shipbuilding and Naval Architecture in Canada *(National Museum of Science, 1994), 60, 61.

10. Ibid., 82, 83.

11. Marc Milner, *Canada's Navy: The First Century*, 2nd ed. (University of Toronto Press, 2010), 304.

12. “Value for money: Economic impact of the Halifax shipyard under the National Shipbuilding Strategy,” Conference Board of Canada, Ottawa, 29 May 2023, 3.

13. John Birkler, et al., *Australia's Naval Shipbuilding Enterprise* (RAND Corporation, 2015), 148–149.

14. Wilson, *A History of Shipbuilding and Naval Architecture in Canada*, 52.

15. Terry Milewski, “Canada's vast shipbuilding plan still at starting line,” CBC News, 4 May 2015, https://www.cbc.ca/news/politics/canada-s-vast-shipbuilding-plan-still-at-starting-line-1.3058147.

16. David Pugliese, “Irving wants to sell Arctic patrol ships to other customers but are the vessels too expensive?” *Ottawa Citizen*, 29 November 2017, https://ottawacitizen.com/news/national/defence-watch/irving-wants-to-sell-arctic-patrol-ships-to-other-customers-but-are-the-vessels-too-expensive.

17. Eric Lehre, “The National Shipbuilding Procurement Strategy: An update,” Canadian Global Affairs Institute, Ottawa, February 2013, 8.

18. Author email correspondence with members of the Canadian Naval Review editorial board, 12 March 2020.

19. “Global combat ship variant selected for Australia's Project SEA 5000 frigate programme,” *Warship Technology* (October 2018): 18.

20. Douglas Fraser, “Steadying the drumbeat,” BBC News, 12 September 2019, https://www.bbc.com/news/uk-scotland-scotland-business-49670332.

21. “National Shipbuilding Strategy: A refreshed strategy for a globally successful, innovative and sustainable shipbuilding enterprise,” UK Ministry of Defence, London, 2022, 32.

22. Birkler, et al., *Australia's Naval Shipbuilding Enterprise,* 96.

23. Lewis Johnman and Hugh Murphy, “The rationalisation of warship building in the United Kingdom, 1945–2000,” *Journal of Strategic Studies* 24, no. 3 (September 2001): 118.

24. Stuart Young and Jonathan Davies, “United Kingdom warship procurement strategies: Accident or design?” in Doug L. Bland, ed., *National Approaches to Shipbuilding and Ship Procurement* (Queen's University School of Policy Studies, 2010), 3.

25. Nicholas de Larrinaga, “UK Type 31 frigates should not be BAE-built, key report recommends,” *Jane's Defence Weekly*, 29 November 2016.

26. John Birkler, et al., *The Royal Navy's New-Generation Type 45 Destroyer: Acquisition Options and Implications* (RAND Corporation, 2002), xx and 77.

27. “National Shipbuilding Strategy: The future of naval shipbuilding in the UK,” UK Ministry of Defence, London, 2017, 23.

28. Richard Scott, “SDSR 2015: Industry waits on national shipbuilding strategy,” *Jane's Defence Industry*, 24 November 2015.

29. RUSI Professor Trevor Taylor, as quoted in House of Commons Defence Committee, “We’re going to need a bigger Navy: Third report of the House of Commons Defense Committee inquiry on The Navy: Purpose and procurement,” House of Commons, London, 7 December 2021, 58. I am grateful to PhD student Mark McWhinney for research assistance in identifying this report.

30. Andrew Davies and Mark Thomson, “An enterprise-level naval shipbuilding plan,” Australian Strategic Policy Institute, 2015, 8.

31. “National Shipbuilding Strategy,” 2022, 31.

32. Milner, *Canada's Navy*, 24.

33. House of Commons Defence Committee, “We’re going to need a bigger Navy,” 66.

34. Commander (retired) Ken Hansen, as quoted in Standing Committee on National Defence, “The readiness of Canada's naval forces,” House of Commons, Ottawa, June 2017, 48.

35. “National Shipbuilding Strategy,” 2017, 12.

36. David Pugliese, “Canada takes modular approach to future vessels,” *Defense News*, 20 October 2008, 14.

37. The ship in question was the ill-fated Littoral Combat Ship. See Emma Salisbury, “Beware the allure of mission modularity,” *US Naval Institute Proceedings* 149, no. 5 (May 2023), https://www.usni.org/magazines/proceedings/2023/may/beware-allure-mission-modularity.

38. Pat Finn, Assistant Deputy Minister (Materiel), DND, as quoted in Murray Brewster, “Cost and Canadian content concerns hover over warship plans,” CBC News, 27 October 2016, https://www.cbc.ca/news/politics/frigate-replacement-program-1.3824931.

39. Author conversation with high-ranking naval officer, 15 November 2021.

40. Tim Fish, “Continuous build: Australia's shipbuilding enterprise takes its next steps,” *Jane's Navy International*, 8 November 2018.

41. Ben Packham, “Dutton sticks to his guns on frigates,” *The Australian*, 2 February 2022, https://www.theaustralian.com.au/nation/defence/peter-dutton-sticks-to-his-guns-on-frigates/news-story/6064692f3cf8be3ccd516f9723d5b493.

42. “It's broke and it's time to fix it: The UK's defence procurement system,” Defence Committee, House of Commons, London, 16 July 2023, 14.

43. “Navy Constellation Class frigate program: Background and issues for Congress,” Congressional Research Service, Washington, DC, 11 July 2024, 7.

44. Andrea Migone, Alexander Howlett, and Michael Howlett, *Procurement and Politics: Strategies of Defence Acquisition in Canada and Australia* (Palgrave Macmillan, 2023), 38, 95.

45. Kym Bergmann, “Hunter Class frigate program could be accelerated,” *Asia Pacific Defence Reporter*, October 2022, 85, https://asiapacificdefencereporter.com/hunter-class-frigate-program-could-be-accelerated/.

46. John Ivison, “Our navy sinks toward impotence because Ottawa can’t manage to procure ships,” *National Post*, 10 January 2024, https://nationalpost.com/opinion/our-navy-sinks-toward-impotence.

47. Auditor General of Australia, “Department of Defence's procurement of Hunter Class frigates,” Australian National Audit Office, Canberra, report no. 21 2022-23, 9.

48. “Naval Shipbuilding Plan,” Australia Department of Defence, Canberra, 2017, 105.

49. “National Shipbuilding Strategy*,*” 2017, 25, 40.

50. “It's broke and it's time to fix it,” 36, 39.

51. David Axe, “The Royal Navy isn’t done yet: Great Britain is replacing its Type 45 destroyer,” *States News Service*, 12 December 2019, https://nationalinterest.org/blog/buzz/royal-navy-isnt-done-yet-great-britain-replacing-its-type-45-destroyer-103912.

52. “$45Bn frigates slow, unsafe: Defence,” *The Australian*, 1 February 2022, https://www.theaustralian.com.au/nation/defence/45bn-hunterclass-frigates-slow-unsafe-defence/news-story/8e7567f0379079f2b1bf4c0a6bb0b989#:∼:text=9%3A53PMJanuary%2031%2C%202022.&text=Australia's%20new%20%2445bn%20Hunter,classified%20Defence%20Department%20report%20reveals.

53. Norman R. Augustine, *Augustine's Laws* (American Institute of Aeronautics and Astronautics, 1983), 53.

54. Jurgen Brauer, Keith Hartley and Stefan Markowski, “Rethinking Augustine's Law; Armament costs and evolving military technology,” in Madhumita Chatterji and Partha Gangopadhyay, eds., *New Frontiers in Conflict Management and Peace Economics: With a Focus on Human Security* (Emerald Publishing Limited, 2021), 3.

55. Migone, Howlett, and Howlett, *Procurement Politics*, 27.

56. Jeffrey F. Collins, “Overcoming ‘boom and bust’? Analyzing national shipbuilding plans in Canada and Australia,” *Canadian Global Affairs Institute Policy Paper*, January 2019, 14.

57. Keith Hartley, *The Case for Markets in Defence: Driving Efficiency and Effectiveness in Military Spending* (London, UK: Institute of Economic Affairs, 2023): 39.

58. Milner, *Canada's Navy*, 215.

